# “The Hard Problem of Consciousness”. Theoretical solution of its main questions

**DOI:** 10.3934/Neuroscience.2019.2.85

**Published:** 2019-05-24

**Authors:** David I. Dubrovsky

**Affiliations:** Department of Theory of Knowledge, Institute of Philosophy of the Russian Academy of Sciences, Moscow, Russia

**Keywords:** information, consciousness, brain, subjective reality, free will, neuroscience, cod, decoding brain neurodynamic codes

## Abstract

The problem of explaining the connection between the phenomena of subjective reality and brain processes is usually called the “Hard problem of consciousness”. The solution of its main theoretical issues is of great importance for the development of modern neuroscience, especially for such direction as neurocryptology (“Brain-Reading”). From the standpoint of the information approach it is proposed to address these issues, namely: 1) the nature of the connection with brain processes of the phenomena of subjective reality (mental state), 2) their causal ability to control bodily functions; 3) the compatibility of freedom of will with the determinism of brain processes and also 4) the frequently asked question: why is information about the acting agent not just represented, but experienced in the form of subjective reality. The connection between the phenomenon of subjective reality and the corresponding brain process is the relationship between information and its carrier; specific features of this relationship are analyzed. Mental causation is a kind of information causality that differs from ordinary physical causation because information is invariant with respect to the physical properties of its carrier, can be coded in different ways. The author identifies and describes those analytical parameters that must be taken into account in the problems of constructing a model of the code neurodynamic structure of the phenomena of subjective reality. A hypothesis is advanced on the emergence of the very quality of subjective reality in the course of biological evolution. On this basis, the methodological aspects of the problem of deciphering brain neurodynamic code of the phenomena of subjective reality and the prospects of neuroscience studies of consciousness are discussed.

## 1. Introduction. Subjective reality as an object of a neuroscientific investigation

In modern analytical philosophy the problem of consciousness is called a “Hard problem” [1],[2] because consciousness has a specific and inalienable quality of subjective reality (let us abbreviate SR). It is this quality that is the main stumbling block for its scientific explanation SR is the reality of the conscious states of the individual, which directly certifies for him that he exists. The quality of the SR is indicated in the philosophical literature by different, but in their meaning similar terms: “mental”, “introspective”, “phenomenal”, “subjective experience”, “qualia”, etc. In recent decades, the term “SR” has become quite widely used to describe the specifics of consciousness, including representatives of analytical philosophy [3]. The concept of “SR” encompasses both individual phenomena and their types (sensations, perceptions, feelings, thoughts, intentions, desires, volitional efforts, etc.), as well as an integral personal formation united by our self, taken in its relative identity to itself, and thus in its reflexive and non-reflexive unity, actual and dispositional dimensions. This holistic formation is a dynamical continuum temporarily interrupted by deep sleep or in cases of the loss of consciousness. SR always represents a certain “content”, which is given to an individual in the form of a “current present”, i.e*. now*, although this “content” can relate to the past and the future.

A specific features of SR phenomena, as already noted, is that physical properties cannot be attributed to them (mass, energy, spatial characteristics): they differ from the subjects of the study of classical natural science and evince a special ontological status, the definition of which has always presented difficult questions to philosophers of materialistic orientation and naturalists, especially for those who studied the connection of psychic phenomena with the activity of the brain.

These complex issues at the ontological level have, on the other hand, no less epistemological questions. The point is that the description of SR phenomena is made in terms of intentionality, purpose, meaning, value, will, etc., while the description of physical phenomena and brain processes—in terms of mass, energy, spatial characteristics, etc., and between these conceptual complexes there is not direct logical connections. Some intermediate conceptual link is required to link, to combine these different types of descriptions in a single conceptual system capable of providing a theoretically grounded explanation of the connection between SR phenomena and brain processes. How to find it and thereby overcome the “explanatory gap”? This is how the representatives of analytical philosophy term the situation in regards to the Mind-Brain Problem.

At the same time, SR represents the “internal”, individual-subjective experience inherent only in a concrete individual (expressed in first person reports). How do we transfer from this individual-subjective experience to intersubjective, generally valid statements (from a third person) and to the substantiation of true knowledge? In general philosophical terms, these questions have been repeatedly posed and variously resolved from one or other classical position. However, in light of the pressing problems of modern science, they continue to be open. This is especially acute in those branches of neuroscience that are aimed at researching mental activity, the phenomena of consciousness, and do not accept reductionist solutions (i.e. concepts that seek to reduce SR phenomena to physical processes, speech or behavioral acts). In this respect, the questions of phenomenological analysis and systematization of SR phenomena, the discretization of the SR continuum, the formation of such invariants of SR phenomena which could serve as sufficiently definite objects for correlating them with brain processes, become of fundamental importance (I will return to this later).

To solve this problem, first of all, a theoretically substantiated answer to two main questions is required:

**1. How to explain the connection between the phenomena of SR and brain processes, if physical properties (mass, energy, spatial characteristics) cannot be attributed to the first, while the latter necessarily possess them?**

**2. If physical properties cannot be attributed to the SR phenomena, how can one explain their ability to causally affect bodily processes?**

In addition to these basic issues, there are a number of others, which usually form stumbling blocks for natural scientists and urgently require solutions. However, we must immediately say that the answers to them are determined by solutions available for the first two. Moreover, it could be argued that they depend more on the solution of the first fundamental issue. These other significant issues are as follows:

**3. How to explain the phenomena of voluntary actions and free will and how to combine them with the determinism of brain processes?**

**4. How to explain the emergence of the very quality of SR in the process of evolution**, which, at first glance, seems to be unnecessary for the effective functioning of the organism (which always served as an excuse for epiphenomenalistic interpretations of SR and reductionist constructions, the use of models of “zombies”, etc.)?

**5. Why is information about the acting agent not just represented, but experienced in the form of SR?** This is a question closely connected with the previous one (it is usually sharply posed by representatives of analytical philosophy). These and a number of other particular questions will be singled out and theoretically interpreted below.

## 2. The proposed theory

It relies on modern knowledge of biological evolution and the processes of self-organization (biological and social, including its technical components) and uses an information approach to address the issues raised [4]–[7].

It should be noted at once that, despite the difference in the philosophical interpretations of the notion of information and the absence of a unified information theory, this concept has generally accepted meanings. I use the concept of information in the general sense in which it is used in practically all sciences, namely: as the “message content”, “signal content” (N. Wiener's definitions). Therefore, there is no need to go into his various philosophical interpretations, to evaluate each of the two basic concepts of information (attributive and functional), to choose one or another. Although I prefer a functional rather than an attributive concept, the information approach to the Mind-Brain Problem developed below is compatible with both. The theory I propose is relatively clear and simple, and therefore it is convenient for criticism. Three initial parcels are accepted in it. The first two are principles with no empirical refutations, the third is an intuitively acceptable agreement, which is convincingly confirmed by ordinary and scientific experience. I quote these initial assumptions.

I. Information must be embodied in its physical medium (does not exist outside and beside it).

II. Information is invariant with respect to the physical properties of its carrier, i.e. the same information (for a given self-organizing system—for a given organism, a person or a community) can be embodied and conveyed by carriers of different physical properties, i.e. encoded in different ways. For example, the information that rain is expected tomorrow may be transmitted in different languages, orally, in writing, using the Morse code, etc.; in all these cases its carrier can be different in mass, energy and space-time characteristics). Let us abbreviate this principle—IP.

III. SR phenomenon (for example, my sensory image in the form of visual perception of some object A, experienced at a given interval) can be considered as information (about this object).

Note that the information allows not only a syntactic description, but also a semantic (content-semantic) and pragmatic (target, value, “effective”, program-managing) description, which meets the requirements for describing the phenomena of SR. If these three initial assumptions are accepted, then the desired explanatory consequences are logically deduced from them. The following is the answer to the t question indicated above.

### 2.1. How to explain the connection between the phenomena of SR and brain processes?

1. Since this phenomenon of SR is information about A (we denote it by **A**), it has its own definite carrier (we denote it by **X**), which according to the data of neuroscience is a certain cerebral neurodynamic system. Thus, the phenomenon of subjective reality is necessarily related to an appropriate brain process as information to its carrier. Although the neurodynamic system **X** necessarily consists of physical components, its functional specificity cannot be explained on the basis of physical properties and regularities (since, as it is well known, the description of functional relations is logically independent of the description of physical properties and relations). This is shown by an analysis of the nature of the necessary connection between **A** and **X**.

2. The connection between **A** and **X** is not causal, it is a special kind of functional connection: **A** and **X** are simultaneous and one-cause phenomena; they are in relation of mutual single-valued correspondence; **X** is the code embodiment of **A** or, briefly, the code **A**. This kind of connection can be called a ***code dependence***, it is formed in the phylogenesis and ontogenesis of the self-organizing system (it has the character of a historical neoformation and in this sense is random, i.e. the information acquired in this self-organizing system has such a code embodiment, but in principle it could have another, but, having arisen in this form, it becomes a functional element of the process of self-organization in this system). This relation is valid, i.e. retains its functional role either in one-time action or in some interval (for example, conditioned reflex communication), and often throughout the life of the individual and even the through the entire history of the species, and in the case of the fundamental DNA code—for the entire period of the existence of living systems on the Earth. But even the genetic code is not an exception, its emergence was not necessary, also had a probabilistic, random character^1^. Even more it is inherent in the origin of the code structure of language (as evidenced by many different languages). However, the random nature of the formation of this code dependence does not cancel the principle of the necessary connection of information and its carrier, but only indicates that the specific carrier can be different in its physical properties (in accordance with the **IP**).

Of course, in the course of evolution, more economical forms of codes were selected for their mass, energy, space-time characteristics. In a complex self-organizing system (that is, consisting of self-organizing elements and subsystems), there is a multi-step hierarchy of code dependencies that distinguish its history (both phylogenetically and ontogenetically). This hierarchy of code dependencies is the main levels and nodes of the organization of the given system and, consequently, the main contours of the management structure. The experience of this type of system testifies to very complex relations of centralization and autonomy in their integral functioning. These relations are still poorly understood. However, there is no doubt that this is a kind of fusion of hierarchical centralization of code dependencies with a high degree of autonomy for certain levels of organization, which includes not only cooperative relations but also competitive ones. Self-organization is a multidimensional dynamic structure of code dependencies (or information processes, respectively). Hence the special urgency of studying the nature of code dependence as an element of self-organization. The connection between **A** and **X**, like any code dependence, qualitatively differs from a purely physical relationship, it expresses the specifics of information processes. Among them, some informational processes in the brain related to the quality of SR are represented in the form of code formations of type **X**. A thorough study of the connection between **A**–**X**, the structural and functional organization of systems of type **X**, means the deciphering of the brain code of this phenomenon of SR.

3. But what does the decoding operation mean, if the information is necessarily embodied in its carrier, and the latter always represents one or other of its code incarnations (i.e. if the information always exists only in a certain code form and nothing else)? It can only mean the transformation of one code into another: conversion of “incomprehensible” for the given self-organizing system into “understandable”. Therefore, two types of codes should be distinguished: 1) “natural” and 2) “alien”. The former are directly “understandable” to that self-organizing system to which they are addressed; more precisely, the information embodied in them is “understandable” (for example, the values of the patterns of the frequency-impulse code, coming from certain structures of the brain to the muscles of the hand, the words of the native language for the interlocutor, etc.).

The information is “understandable” in the sense that it does not require a decoding operation and can be directly used in order to ensure management. The “natural” code carries information in a form open to “understanding”; it does not require either a study of the structure of the signal nor special analysis of the information. We perceive a friend's smile not as a set of movements of a multitude of facial elements, but immediately in its integral “meaning”. Unlike the “natural” code, “alien code” is directly “not understandable” for a self-organizing system, it cannot perceive and use the information embodied in it. For this, it needs to perform a decoding operation, i.e. a transformation of the “alien” code into “natural” code. It is important to note that in cryptology and after it in modern science the term “code” is usually not used to denote objects that have “natural” codes (because of their “transparency”). However, the approach I propose to decipher the cerebral codes of SR phenomena relies on a broader theoretical basis in comparison with classical cryptology, in which a narrow interpretation of the concept of code is adopted^2^. The way to convert “alien” code into “natural” code is either initially programmed in the structure of a self-organizing system, or it was created on the basis of its experience and as a result of random findings. Often it remains unknown to us and might be researched by a researcher due to persistent search (as evidenced by the experience of cryptology, linguistics, ethnography, other sciences where such a problem arises^3^).

4. Both “natural” and “alien” codes are for a given self-organizing system (organism, its subsystems, personality, community etc.), internal and external. Apparently, the “alien” codes are mostly external. However, they also exist at the level of the individual in the processes of autocommunication. Here, the internal “alien” codes are manifested in the form of incomprehensible and often negative in their “meaning” subjective experiences and symptoms that take their origin in unconscious and somatic sphere; this also applies to a variety of cases of psychopathology. Let's pay attention to the apparently paradoxical situation: the code of type **X** is for me an internal “natural code” in the respect that directly opens to me the information contained in it (i.e. the image **A**). In this respect, the code **X** is decoded in my brain automatically. But at the same time it is for me an external “alien” code in the sense that I do not know anything about its location in my brain, its composition and functional structure (and generally do not feel what is going on in my brain while I am experiencing the image **A**).

In other words, in the SR phenomena I have the information in its “pure” form, and the information about its carrier is completely closed. However, in order to understand the specific dependence of **A** on **X**, it is necessary to know the structure of this carrier, it is necessary to decipher its code structure, just as it is required in learning a previously unknown language. Here **X**, being for me and to all of us an “alien code”, becomes a special object of investigation with the aim of deciphering it, clarifying the information contained in its **A** in an independent way, i.e. based on the removal of signals from my brain and with the help of certain methods of converting **X** to a suitable “natural code” (in the form of text, image, digital record, etc.) that is always automatically converted to an internal “natural code” of the researcher's brain which serves a guarantee of content understanding in this information (in the form of the corresponding phenomena of SR). And this provides an understanding of the results of deciphering the code **X** by other researchers and other people, i.e. its intersubjective status^4^.

Thus we can speak about the possibility of the emergence of a new type of communication, which can now serve as an object of serious philosophical reflection on the future of terrestrial civilization. Along with the development of brain-computer interfaces, on the basis of which significant results have been achieved, there is the task of creating a so-called “neuronet”, i.e. of “brain-brain” interfaces, which promise us the creation of a fundamentally new type of communications. If the brain codes of SR phenomena are thoroughly deciphered, this will violate the fundamental principle of social self-organization—relative autonomy, “closeness” of the subjective world of the individual. What will happen if it is “discovered” beside the will of its owner, if some become “open”, and others “closed”, etc.? No less interesting is the question: what will happen with our society, with its political, economic and other institutions, if all modern homo sapiens and institutional subjects suddenly become “open”? (Nobody can deceive anyone, everyone tells only the truth; let's conduct such a thought experiment).

5. Accordingly, two different types of codes (“natural” and “alien”) and two different aspects of decoding the code should be distinguished. When decoding an “alien” code (that is, transforming it into “natural” code), the task is to understand its information content. Conversely, when deciphering a “natural” code, the structure of which is unknown, the task is to recognize, understand precisely its structure (structural-functional-spatial-temporal characteristics, physico-chemical organization). Hence, there are two types of code decoding tasks: direct and reverse.

Direct task: a code object being given, the task is to find out the information contained in it. In the case of code objects of type X, difficulties are related to determining and making a description of it, not to mention the search for ways to decode the code and implement the decoding process7. Reverse task: information is given (say, A, i.e. the information in a “pure” form), it is required to determine its carrier and to study its functional structure in order to independently reproduce this information. Due to **IP**, this task is more difficult than the direct one, since the given information may have different carriers (although their diversity is limited by the properties of the brain, by the specificity of its substrate, elements, synaptic connections, morphological structures, etc.).

To this we should add that any translation of the information into another language entails some loss of the original content (a question requiring special analysis). In the real process of studying code dependencies, direct and reverse tasks reveal a close interdependence. Nevertheless, in the problematic of deciphering the neurodynamic code of psychic phenomena, the reverse task occupies a dominant position, for here the search is directed from the information given to us to its carrier. In the case under consideration, from **A** to the desired neurodynamic correlations, which should correspond to varying degrees to **X**. These correlations are established and investigated in modern neuroscience using various methods (EEG, EMG, fMRI, PET, etc.). In this case, the detected correlates are only indirectly associated with X, which represents an extremely complex, multidimensional circular neurodynamic network system, and require special analysis and interpretation using mathematical and other means to construct adequate models of the desired code dependence.

Over the past ten years, great results have been achieved in deciphering the brain codes of visual perception, not only in the case of static and relatively simple black and white visual images8, but also in the decoding of moving color images—a fragment of a movie perceived by the subject (corresponding images experienced by him, reproduced on the computer screen as a result of analysis and synthesis of elements of their brain correlations received mainly through the use of the fMRI method (see: [8],[9]). This direction of neuroscience, which is called “brain-reading”, or more accurately could be named neurocryptology, is developing rapidly and sets the task of deciphering the brain codes of various phenomena of SR (not only visual, but auditory and tactile perceptions, emotions, voluntary actions and even thinking^5^). It acquires a strategic importance for the creation of new “brain-machine” interfaces and the development of convergent technologies (NBIC). To increase the effectiveness of this area of neuroscience research, however, a thorough phenomenological development of code deciphering objects is necessary, i.e. articulation and formation of sufficiently defined phenomena of SR. In existing studies, the decryption object of the code (i.e., the distinguished phenomenon of SR) remains largely undetermined, which reduces their effectiveness.

6. Forming the code decryption object requires a correct sampling of the continuum of the SR as a “current present”, breaking it into specific elements and fragments. Where it is possible, it is desirable that sampling reach the level of quantification of SR phenomena. Such an operation can be realized for relatively simple phenomena of SR (sensations, perceptions, some emotional states). It involves minimizing the given phenomenon of SR by “content” and in time. For example, in the tachistoscopic experiment, I perceive a white square on a black background in a dark room for a minimum time. This can be called a quantum of visual perception. We agree that the information A, discussed above, is just such a quantum of SR. Then its carrier **X** is the desired code structure **A** (at least, the observed neurodynamic correlation **A**) should be limited to the same time interval. The set of such my perceptionquanta makes it possible to form a personal invariant of perception (information) **A** and thus to assume the corresponding personal invariant **X**. In the same way, one can also form interpersonal invariants **A** and **X**, when the participants of the experiment are different individuals. Clear invariants of this kind are needed to comply with the principle of repeatability of the experiment. This applies not only to invariants of certain types of phenomena of SR, but also to the invariant of any phenomenon of SR, i.e. to the invariant description of any realized state in general, and accordingly to the description of those specific properties of brain activity, those specific information processes that determine the presence, in the given interval, in all of us the quality of SR (in contrast to information processes in the brain, which, according to D. Chalmers, “go on in the dark”).

Despite the **IP**, there are sufficient grounds to believe that code-based neurodynamic systems of type **X,** which are carriers of certain phenomena of SR, although they include a wide range of elements and properties, nevertheless have essentially general characteristics that allow us to determine and decipher the code of this information (of this SR phenomenon). One usually refers to the fact that the observer is dealing with individual, original, unique phenomena. But he, one way or another, always overcomes this abyss of diversity, creating suitable invariants. A necessary condition for scientific research is the formation of such invariants, which register unity in diversity, and their use for the purpose of scientific explanation. It can be said that this is a common place for a scientist. But, like many simple truths, it contains a lot of theoretical difficulties that particularly affect the problem of deciphering the brain codes of psychic phenomena and, first of all, in solving the problems of forming clear 0 invariants of those SR phenomena that are offered as an object of deciphering their brain codes^6^.

These difficulties are exacerbated by the absence of taxonomy of SR phenomena, the shortcomings of their classification, the extreme weakness of attempts to theoretically order their diversity. This applies even more to the understanding of the extremely complex, multidimensional value-semantic and active-volitional structure of the SR and its self-organization, the core of which is our “I”. In the meantime, every single phenomenon of the SR, even in the form of its personal invariant, always carries in itself to some extent the properties of this structure and cannot be comprehended outside of these properties. Hence, it follows that the task of deciphering the neurodynamic code of a given SR phenomenon must register these properties.

7. As it was shown above, **A** and **X** are simultaneous, one-cause phenomena that are in a single-valued correspondence. But this means that the phenomenological description of the essential properties of **A**—at least of its formal content, temporal, structural, dynamic properties—can be extrapolated to **X**, i.e. can serve as a primary model of **X**, pointing to the essential properties of **X** that are necessary for understanding its code organization. Let's try to distinguish these properties, i.e. basic parameters of the description of any phenomenon of SR. The processes of coding and decoding primarily indicate the necessary participation in them of memory, they indicate the circular structure of actualization-deactualization of the SR's experienced content in this interval of the “current present”. These aspects of code interpretation are of fundamental importance and are the subject of special studies.

We shall concentrate on the phenomenological properties of the code decoding object (of the isolated SR phenomenon), which are caused by the multidimensional dynamic structure of SR. In each SR phenomenon, a mapping is shown not only of some “external” content, but also of it itself. This is manifested in its irremovable belonging to its “own” I (which in psychiatry is called the “sense of belonging”, it is broken only in pathological cases, and implies the phenomena of depersonalization well described by psychiatrists and usually associated with them the phenomena of derealization). This unity of external-imaging and self-imaging allows us to consider that the basic dynamic structure of SR is bimodal, i.e. its main introsubjective relations, which determine the dynamic integrity of the SR, represent a unity of the opposite modalities of “**I**” and “**not**-**I**”, which is realized by their mutual position and variable correlation^7^. Such bimodality, including the mechanism of variable correlation of the mirror type, must also be inherent in the neurodynamic organization of the code structure of any SR phenomenon. Now we are far from understanding its “device”, but it is this feature of every act of consciousness that relieves us of the infamous homunculus and allows us to explain the phenomenon of mapping the mapping (information on information) that is characteristic of any phenomenon of SR. In addition to the above two integral parameters (memory and basic structure of SR), six more parameters for describing the phenomenon of SR taken for the purpose of deciphering its neurodynamic code must be singled out. They can be called analytical, since each of them denotes one “dimension” in the multidimensional dynamic structure of the SR. Taken together they serve to describe a model capable of displaying the essential properties of the desired codal neurodynamic organization.

8. Let's consider each of them.

1) **The time parameter**, which has already been mentioned above, fixes the selected phenomenon of SR in a certain time interval. In the same interval, its neurodynamic code also functions, which limits the zone of its search and identification.

2) **The content parameter** (or, rather, the parameter of content) means that any SR phenomenon is a mapping and the meaning of something. This is the “content” of a certain interval of the “current present”, regardless of its adequacy or inadequacy, and whether it acts as a “one-time” experience of a given individual or in the form of a personal invariant, an interpersonal invariant or in any other different form. This parameter indicates the register of the neurodynamic organization through which the “content” of this phenomenon is coded, and aims at an experimental search for this register (functional mechanism). The latter presents, apparently, the greatest complexity in the problem of decoding the code. Although simple types of “content” of SR phenomena are reproduced on the computer screen using the fMRI method, we are aware that the tomogram observed in the brain, being a correlate of the SR phenomenon experienced, nevertheless very indirectly expresses its real, codal neurodynamic organization. This is, so to speak, only the first step in solving the problem of deciphering brain codes of SR phenomena.

3) **The formal parameter** means that any content of the phenomenon of SR appears in a certain form and refers to the corresponding class, gender, species, i.e. it is somehow categorized. When we talk about visual perception or perception in general, we have in mind a certain form of existence of sensual images. It orders their colossal variety. Despite the absence of a scientific taxonomy of SR phenomena, we mostly successfully use formal discretizations, which are set by psychology on the basis of generalizations of everyday experience, natural language (sensation, visual perception, perception in general, representation, concept, etc.). The formal parameter registers the necessary property of the SR phenomenon and therefore compels the introduction of this parameter into the model of its neurodynamic code organization; it aims at elucidating those functional neurodynamic mechanisms that perform operations of categorization, classification, generalization, identification.

4) **The truth parameter** characterizes any SR phenomenon in terms of the adequacy of the mapping of the corresponding object. It can be true or false, questionable or indefinite. However, in all cases, we still have a fundamental attitude toward truth and rightness, which functions disproportionately and is often areflexive. We are constantly “tuned” to achieve adequate knowledge of what interests us. Any interval of the “current present” includes the authorizing register of “accepting” or “not accepting” this “content” (including doubt, probabilistic assessment, feeling of uncertainty). It is far from perfect and often selects false and ridiculous ideas as “true” and “right”. However, all the really true ideas and theories that originated and began their journey in the minds of individuals were sanctioned in the beginning by a personal register. and only with time did they receive confirmation at the level of interpersonal and suprapersonal social-register sanctioning. The presence of a personal authorizing register in the structure of SR phenomena allows us to assume the same kind of functional mechanism in the neurodynamic code organization of the SR phenomenon under study and to outline the ways of its special investigation.

5) **The value parameter** characterizes the significance for an individual of the “content” of the experienced SR phenomenon, its relation to this “content”. The value “dimension” of the SR has a specificity that is not reducible to the “truth” and other parameters of the SR. It is well known that false representations can have an extremely high value for a person, and true ones a very low and even negative significance. In this respect, the value parameter, like the truth one, has two poles, one of which expresses a positive value and the other a negative one. The structure of personal attitude values includes three main types: 1) hierarchical (a clear distinction between higher and lower values, subordination of the lower to higher, unambiguous choice); 2) rank-and-file (when numerous value intentions are located at approximately one, mostly low level, they are easily interchangeable and the choice between them is either extremely difficult or, conversely, very simple); 3) competitive (when two value intentions are incompatible but a choice is required, if it is not produced it generates an agonizing state of ambivalence, which, however, can be successfully overridden). It is the value intention that dominates a given time interval and determines choice, decision and action. The value parameter denotes a similar specific functional register in the code brain organization of SR phenomena that implements motivational stimuli and various types of sanction (cognitive, emotional, painful, etc.

6) **The activity (intentional-volitional) parameter** characterizes any phenomenon of SR in terms of its activity, highlighting such factors as future projections, probabilistic predictions, goal-setting and purposefulness, volition, action and creative new developments. This parameter expresses an activity vector as a special quality that cannot be replaced by any of the above parameters despite a close connection with them, especially with the value parameter. It is important to consider the activity in its self-development as a process of new formations, including significant changes in its direction and ways of realization, as an opportunity for the formation of increasingly perfect forms of activity. This parameter aims at the study of those specific functional mechanisms in the brain that support active states and implement them in various activities. Its clear awareness stimulates the study of the processes of neurodynamic self-organization, which serves as an indispensable factor in the functioning of neurodynamic code carriers of SR phenomena. Briefly outlined above, then, are the two integral and six analytical parameters indicating those analogous functional registers of the brain neurodynamic organization that should serve as the object and purpose of neuroscience research in solving the problem of deciphering brain codes of SR phenomena. Registering the main dynamic measurements of the multidimensional structure of SR, they can be used to construct more advanced computer models of code representation of SR phenomena in the brain, and thereby to understand the dynamic self-organizing structure that functionally determines the quality of the SR.

9. Proceeding from the principle of invariance of information (IP) in relation to the physical properties of its carrier and, accordingly, the principle of system isofunctionalism (substantiated by A. Turing)^8^, one can draw a conclusion about the theoretical conceivability of reproducing the quality of SR on other, non-biological substrates. SR is a functional property of the neurodynamic self-organizing system. There is no theoretical prohibition on the realization of this property on other suitable substratum bases. It is possible to create such elements (differing from neural ones in physicochemical and morphological features) and a dynamic self-organizing system built from them that will be able to reproduce information processes that determine the quality of SR, i.e. to represent for the control center of this system an information in a “pure form” and the ability to operate on it, and thus to constrain the reflexive and bimodal registers of information processing characteristic of ourselves. In this direction, the convergent development of NBICS (nano-technologies, bio-technologies, information, cognitive, social technologies and corresponding scientific disciplines), which is creating new components and ways of self-organization, opens new perspectives for the formation of artificial intelligence and for the transformation of human nature. In recent years, these issues have become the subject of thorough discussion by major scientists and philosophers. It is of strategic importance, determining the future of mankind and the direction of anthropo-technological evolution, and, above all, represents the way out of the steadily deepening global crisis in our consumer civilization.

We now turn to the answers to the second main question.

### 2.2. If physical properties cannot be attributed to the SR phenomena, how can one explain their ability to causally affect bodily processes?

1. The phenomenon of SR causes external or internal bodily changes, complex actions of the personality, and determines their result as information on the basis of the existing code dependence, which is a kind of “isolated” information in the continuum of physical interactions (as long as the code structure of this self-organizing system is preserved). When I say to a student: “Come up to me!” and he performs this action, it is caused and determined not by the physical properties of the words I uttered but namely by the information expressed with their help, its semantic and pragmatic features. In themselves, the physical properties of the information carrier do not explain the resulting effect, although they necessarily participate in the act of determination. This is confirmed by the fact that I can cause exactly the same effect to be brought about by other words and, in general, by signals very different in their physical properties (by virtue of the principle of invariance of information with respect to the physical properties of its carrier—**IP**).

2. Here we have a special type of causality—information causation. Its specificity in comparison with physical causality is determined by the **IP**. Psychic causation is a kind of information causality; in analytical philosophy it is called mental causation. The notion of psychic causality encompasses also the unconsciously produced actions. Stressing the intimate relationship between the conscious and the unconscious levels of the psyche, we are still primarily interested in consciously assumed actions (which are initiated by the on-going phenomenon of SR, because there are also unconscious psychological forms of causation). Therefore, in this case it is perhaps better to use the concept of mental causality as a subspecies of information causality (if the mental is limited to us by SR phenomena).

3. It is important to emphasize that the concept of information causality does not contradict the notion of physical causality. Physical causality retains its entire significance if it does not pretend to be a universal means of explaining all the phenomena of reality without exception, for example, explaining the causes of the economic crisis or the causes of self-sacrifice of the individual. Psychic causality gives a scientifically grounded answer to the classical question of the impact of the mental on the physical. But here the reciprocal question arises: the effect of the physical on the mental. Even if we disregard those cases where severe mechanical, temperature and radiation-related influences, etc. destroy brain code structure and biological organization, physical cause can serve to explain the essential properties of the mental when it comes to direct sensory mappings (sensations, etc.), or the influences on code structures that lead to mutations, or electromagnetic, chemical (through blood) and further effects on the brain. But even here, physical causes are often mediated by information processes and, accordingly, informational causes. The concept of information causality substantially expands the theoretical means of scientific explanation; it becomes necessary when the subject of research is self-organizing systems (biological, social, and in some respects technical ones). Theoretical and empirical justifications of information causality differ significantly from the principles of description and explanation of physical causality. This determines the ontological status of information (in particular, mental) causality.

We now turn to the third question.

### 2.3. How to explain the phenomena of voluntary actions and free will and how to combine them with the determinism of brain processes?

1. Together with the ability to have information in a “pure form” we are given the ability to operate it across a fairly wide range. This expresses the activity of SR. It includes voluntary actions that can take place not only in the purely mental plane, but also in the communicative and practical ones. An analysis of the structure of a voluntary action indicates the essential role of the areflexive and dispositional levels in it. However, the initiator and regulator of this action is always the specific phenomenon of SR. Therefore, a voluntary action is visual evidence of mental causality. Let us take a simpler example (in comparison with the one given in 2.1). I want to turn on the light of the desk lamp and I do this by pressing the button. In this case, the mental reason in the form of my desire and motivation is a program of actions which triggers a chain of code transformations that have been well worked out in phylogenesis and ontogenesis (i.e., sequential and parallel inclusion of the code programs of the arm movement and other bodily changes associated with it, programs of energy supply of the whole complex of actions leading to the achievement of the goal). The phenomenon of SR, which has a higher value (and belief) ranking, can also have a more powerful causal effect on bodily processes. Well known are the somatic effects of the “overvalued idea” and many such manifestations of the extraordinary power of mental causation, mental control. We may refer to the experience of Second World War with its many striking examples of strength of spirit and will, outstanding feats for the sake of the Motherland, duty, honor, justice, etc.

2. Mental causality means not only the influence of the mental on the corporeal, but also (it is not always taken into account) the influence of the mental on the mental. The fact that one thought can influence another, that it can cause another thought, is the universal fact of our psychic experience. Despite the difficulties involved in discretization of SR phenomena, in relatively simple cases it is possible to fairly clearly represent the associative transition from one of them to another as a cause-effect relation. For example, the visual image of **A** evokes for me, in the next instant, the visual image of **B**. Such mental causality or the “mechanism” of posteriority of **B** from **A**, does not fundamentally differ from those processes when the phenomenon of SR causes a certain bodily change. Only the contours of code transformations, those subsystems of the brain in which they occur, and the character of the effector changes (their presence or absence in external organs) are different.

3. But the information **A** is embodied in the neurodynamic system **X**, and **B** is embodied in the neurodynamic system **Y**, respectively. The transformation of **A** into **B** is the transformation of **X** into **Y**. If I can do it of my own free will, then I can operate and control these brain neurodynamic systems. Managing one's SR phenomena, one's thoughts (as per 1.2) is the management of the corresponding brain code structures. Each of us voluntarily manages a certain class of brain neurodynamic systems (often in not the best way for the self), although this is not sensed and this ability of the self is generally not recognized.

4. But what is our self from the standpoint of neuroscience? According to modern studies (Damasio [10], Edelmen, Tononi [11], Matyushkin [12] and others), our self is represented in the brain by a special structural-functional subsystem, which is called the Ego-system of the brain (or the Self). It includes the genetic and biographical levels of the dispositional properties of the individual and forms the highest, personal level of brain self-organization and management that forms the conscious-unconscious contour of mental processes16. It is at this level that the code transformations that represent our self and information in a “pure” form (that is, as SR) are performed, and ensure the activity of the self in the form of voluntary actions, the self's ability to self-organize, the ability to maintain identity, aspirations and target vectors. The Ego-system embodies the personality characteristics of the individual, the individual's ability to express his will. And here the question arises as to free will and its compatibility with the determinism of brain processes.

5. There is no room here to analyze the problem of free will. But I must say that anyone who denies free will, like unrelenting physicalists, disclaims themselves as persons, absolving themselves of all responsibility for their actions, including the assertion that there is no free will. Each of us is sure that in many cases, at will, at one's own will, one can make a choice and operate with this or that idea, thought or intentional vectors, etc., although in the composition of SR there are also classes of phenomena that are irresistibly imposed on us from the outside or from within our inner world, which are not manageable or only partially manageable, often with great difficulty (pain, emotion, etc.). Nevertheless, our self can control itself and its own phenomena of SR across a very broad range (and indeed expand it). The assertion of the existence of free will has to be drawn from particular cases. But this is quite enough for its recognition (see Dubrovsky [13]).

6. If the ability to voluntarily control one's ideas and thoughts is the ability to control their brain code carriers, this means the ability: 1) to manage the energy supply of these operations, including the corresponding biochemical processes; 2) to change the program of actions, therefore to change their neurodynamic code structures; 3) to expand the contours of mental regulation (including the creation of access to vegetative functions, as yogis do when they, for example, they change their heart rhythms through their volition). This approach allows us to investigate more deeply the phenomena of “exertion of thought”, “exertion of will”, ways of intensifying the creative process, creating new resources for mental self-regulation, not only in terms of the functional but also in terms of the moral. In other words, we are capable of constantly expanding the range of possibilities for managing our own brain neurodynamics (with all the desirable and perhaps undesirable consequences involved).

7. But my ability to voluntarily control my own brain neurodynamics means that the Ego-system of the brain is a self-organizing, self-controlled system. Consequently, the act of free will (in terms of both the produced choice and the generation of internal effort to achieve the goal, including the energy supply of the action) is an act of self-determination. This means that the concept of determination should be taken not only in the sense of external, but also in the sense of internal determination given by the programs of the self-organizing Ego-system and the brain as a whole. Thus, the thesis of the incompatibility of the concepts of freedom of will and the determinism of brain processes is eliminated, and with it the infamous homunculus. These questions are of fundamental importance for deciphering brain codes, since the latter are also self-organizing systems, the functional elements of the brain's Egosystem.

8. In the context of problems of mental causation and free will, a sacramental question often appears, implying an explanatory impasse: how can the mental (SR phenomenon) act on the brain if it is generated by the brain? From the standpoint of an information approach, it is not difficult to answer this. Of course the mental affects the brain in the sense that the activated neurodynamic code system, carrying the personality information in its “pure form” (the mental,) is able to affect other code structures of the brain, including those that carry information processes on the pre-psychic level (i.e. those information processes that are going on “in the dark”), and thereby affect different levels of brain activity, including circulatory processes, biochemical and electrical changes in individual neurons and synaptic networks. This happens sometimes in a particularly strong form. Suddenly a fortuitous thought comes about: Illumination. Emotional outburst. Stormy productive activity—mental or practical. This is an extremely valuable mental state. Initiated at the level of the brain's Ego-system, it produces functional changes in other brain subsystems and, as a result, causes strong reactions in a number of internal organs and throughout the body system. Every mental state of the individual is the product of the specific activity of the brain at the level of its Ego-system, and when it is actualized, the functioning of all brain subsystems essentially changes (in comparison with those states when there is no SR—during a deep sleep or in a temporary loss of consciousness).

There remains one more, perhaps most difficult and hard question—about the origin of the quality of SR. It is equivalent to the question: “What is subjective reality for?”, why did it arise in the course of biological evolution? I will try to answer this briefly from the standpoint of information and evolutionary approaches (more detail see: Dubrovsky [14]).

### 2.4. How to explain the emergence of the very quality of SR in the process of evolution?

1. The process of the emergence of multicellular organisms advanced the cardinal task of creating a new type of management and maintaining integrity, on the solution of which their survival depended. After all, the elements of such a self-organizing system are separate cells, which also represent selforganizing systems with rather tough programs, their having been “developed” by evolution for many hundreds of millions of years. But now the latter had to be coordinated with the general organizational program, and vice versa. This is a very difficult task, the solution of which presupposed finding the optimal measure of centralization and autonomization of control loops, a measure that could ensure the preservation and strengthening of the integrity of a complex living system in its continuing interactions with the external environment. This means a measure of centralization of management that does not violate the fundamental programs of individual cells, and such a measure of autonomy of their functioning that does not prevent, but rather promotes, their friendly participation in the implementation of programs of the whole organism. Together with the centralization of management, it was required to ensure its high-speed efficiency. This measure of centralization and high efficiency was achieved due to the emergence of psychic control in those multicellular organisms that moved actively in the external environment and in ever-changing situation. In organisms with minimal motor activity that are attached to one place, such as plants, the psyche does not develop.

2. Evolution demonstrates the intimate relationship between motor and mental functions, which confirms the emergence and development of the psyche in precisely those complex organisms that are actively moving in the external environment. Hence the obvious causal capacity of the mental (the information in the form of SR,) to directly and instantaneously produce external actions and control the organs of motion Fundamental results on the organic connection between perceptual and motor functions have been obtained in recent years on the basis of the study of “mirror systems” of the brain (see: Rizzolatti, Sinigalia [15]). In contrast to this, the management of internal organs and processes is performed automatically, on the unconscious and pre-psychic levels. At the same time, there is a constant “adjustment” of certain parameters of local and integral changes (energy, information) in the internal environment of the organism for the effective implementation of its actions in the external environment.

3. Psychic control is associated with the process of specialization of cells and the emergence of the nervous system, which performs the functions of programming and implementing actions based on the analysis and integration of information coming from the external and internal environment of the body. Products of this integration are expressed initially in the form of sensations-emotions and only at subsequent stages of evolution, in more complex forms of SR (perceptions, representations, concepts, mental actions, etc.). Accordingly, operational registers of the SR are also complicated. The psyches of animals possesses the quality of SR, which at high levels of evolution takes on a rather complex structure, includes the hierarchical centralization of SR phenomena, i.e. a kind of “Self”, the evolutionary premise of the human Ego-system. The quality of SR represents a specific level of information processes at the level of the Ego-system of the brain.

4. In order for information to acquire the form of SR, its two-step code transformation is necessary at the level of the Ego-system: the first of them presents information to it, that is in the “darkness”, the second forms the “natural code” of higher order, thus creating the phenomenon of information about information, i.e. it is “opened up” and made relevant for the Ego-system, the individual. This is what was called the information given to us “in its pure form” and the ability to operate with it. The state of SR initiates a new type of active occupation in the living system. This is a state of awareness, attention, alertness, constant readiness for immediate action, the state of finding the necessary means of subsistence, probing danger and realizing vital functions. The quality of the SR created by the subsystem of “natural codes” of the second order within the framework of the Ego-system is the quality of virtual reality, its original, fundamental form that acquires, in the process of anthropogenesis, in the emergence of language and in social development, newer and newer forms of external objectification. The processing of information in such a code structure, i.e. at the virtual level, is of high operational efficiency and can be carried out autonomously by external effector functions, which are included only once the program of action has been formed and authorized.

5. The development of the psyche initiated growth of the multistep and multifaceted production of information about information. The range of virtual operations expands, making the generalization of experience more efficient; developing the ability of “delayed actions” and virtual trial actions, the ability of forecasting, and the ability of building models of a probable future; creating an ever-higher level of stirring activity; and multiplying its degrees of freedom. In humans, unlike in nimals, information processes that determine the quality of SR acquire new essential features thanks to the emergence and development of language. This primarily concerns an additional and very productive level of coding and decoding, created by the language system, which qualitatively improves the analytical and synthetic abilities of information operation and develops metarepresentation and reflection.

6. The foregoing contains in many respects an answer to question 5: why the information, about the acting agent, is not simply represented but is experienced in the form of SR? Because the experience in the form of SR combines the functions of mapping and control, it is such a way of “representation” and actualization of the information for the “Self”, which allows it to easily, quickly and, most importantly, voluntarily operate on the information in a “pure” form (i.e at the level of virtual reality). The question “How (through what mechanisms) do information processes in the brain create the quality of SR?” belongs to the competence of modern neuroscience. Studies show that the condition for the emergence of subjective experience is the circular process and the synthesis of information in certain brain structures. Subjective experience in the form of sensations arises when two types of information are compared and synthesized on neurons of the projection cortex of the brain: sensory information (about the physical parameters of the stimulus) and the information retrieved from memory about the significance of the signal. Information synthesis is provided by the mechanism of returning impulses to the places of initial projections after a response from those brain structures that carry out the processes of memory and motivation. Sensation is an act of “information synthesis” [16]; performed within the framework of this cycle; it arises as a result of the high-frequency cyclic process of “self-identification” [17].

7. In any phenomenon of SR given information about some object and information about this information (at least in the form of a sense of belonging to me, to my Self), but as was already noted, absolutely no information is furnished about its brain carrier. Elimination of the mapping of the brain information carrier is inherent in all mental activity. The ability of such a mapping did not arise and did not evolve in the course of evolution due to the **IP**. Since the same information can be embodied and transmitted by media of different properties, the ability to display the medium did not matter for the adequate behavior and survival of the organism. To do this, it needs the information itself (about external objects and situations, about the most likely changes in the environment and about how to interact with it, about one's own states, etc.), the ability to operate it and to use it for management purposes. Exactly these functions have been developed in the process of evolution and anthropogenesis. Humans, while conducting practically all forms of social functions required by life, have no need for the information about brain carriers of that information that they operate.

However, the situation has recently begun to change. Following the decoding of the genome, the problem of deciphering brain codes of psychic phenomena (primarily SR phenomena) has been placed on the agenda, and, as was already noted, it is being successfully pursued. There are reasons to believe that the tasks associated with this problem are caused by the essential needs of the society and that the successes in their solution mark the beginning of a new stage in human development and social self-organization as a whole. Already an elementary analysis shows that the ability of a self-organizing system to display a carrier of information and to control this carrier is unusually expanding the scope of its cognitive and transformational activity and, most importantly, the possibility of self-transformation.

In this regard, we can talk about new opportunities for transforming those genetically conditioned properties of human nature and consciousness which serve as the initial reason for the steady deepening of the ecological crisis and other global problems of terrestrial civilization. It is primarily about the indefatigable consumer intentions of the social individual and his aggression toward to his own kind (and thus toward himself). Of course, there are serious doubts about the possibility of changing these negative properties of human nature that remain within the framework of our biological organization. But if these properties cannot be changed, an anthropological catastrophe awaits us. Of course, other variants of overcoming the present crisis of our civilization and the ascent to a new stage of it are theoretically conceivable, but all of them are somehow connected with such self-transformations that imply a change in the consciousness of the social individual. The latter, to some extent and in certain respects, turns out to be dependent on the results of the development of the Mind-Brain Problem.

## 3. Conclusion

The proposed solution of the main theoretical questions of the “Hard problem of consciousness” can be useful for the development of modern neuroscience studies of the phenomena of consciousness, especially for such a direction as Brain Reading. This concerns the following tasks: 1) the formation on the basis of a phenomenological analysis of the object of investigation, i.e. personal and interpersonal invariants of certain phenomena of subjective reality, with which neurodynamic correlations are established; 2) the solution of a number of methodological issues of the procedure for deciphering the brain neurodynamic codes of these phenomena of SR (they are set out above); 3) refinement of the parameters of the model of the neurodynamic code, which must be taken into account when planning such studies. It should be borne in mind that the neurodynamic correlate of this phenomenon of subjective reality, determined with the help of appropriate methods, reflects only a particular aspect, fragment, a sign of the real neurodynamic code structure, which according to modern views is an activated multidimensional neural network and is cyclical. The proposed theory can contribute to further research of the current theoretical and methodological issues of modern neuroscience—an important condition for its achievement of new frontiers in the study of consciousness.

## Footnotes

1 As one of the pioneers of the genetic code F. Crick testifies, “the genetic code could have almost any structure, since its details depend on which amino-acid and which adapter correspond to each other. Perhaps the existing version of this mutual correspondence was determined at a very early stage of evolution and, probably, the choice in its favor was an accident” ( F.Crick. The Mad Pursuit, A personal view of scientific discovery, Izhevsk, 2004, Pp. 104).

2 In this respect, I conducted a detailed analytical discussion and substantiation of the concepts of code and code dependence (see: Dubrovsky D.I. Information, Consciousness, Brain, Moscow, Higher School, 1980. Chapter 6. Pp. 214–273). The concept of decoding codes, which includes the consideration of methodological issues that relate not only to the Mind-Brain Problem, but the tasks of this kind of research in various sciences, is presented in detail in a special article (see: D.I Dubrovsky. Decoding of Codes: Methodological Aspects of the Problem, in: Voprosy philosophii, 1979, No 11. Reprinted in the book: [6]. Special questions related to deciphering the brain cod see; D. I. Dubrovsky, The Problem of the Neurodynamic Code of Psychic Phenomena (Some Philosophical Aspects and Social Perspectives), Voprosy philosophii 1975, No 6).

3 The experience of solving secret ciphers is very interesting in this connection (see: Simon Singh, Code Book: The Secret History of Codes and Their “Hacking” (Moscow, AST, 2007). Especially instructive is the story of deciphering Maya language by Yuri Knorozov, who did not have a key like the “Rosetta Stone”, which was found by Champollion when deciphering ancient Egyptian cuneiforms.

4 Let us note in this connection the idea of **autocerebroscope**, according to which I myself can observe and study the connection of my own mental and brain processes. In today's conditions, it can have a certain experimental perspective. But even in this case, in spite of my experience of A in a “pure” form, I will have to do the same as an external observer, i.e. get A (its “content”) in an independent way.

5 See: Ivanitskiy A.M. “Reading the Brain”: Achievements, Perspectives and Ethical Problems, Journal of higher nervous activity, 2012. T. 62, No. 2. Pp. 1–10. Due to the fact that the development of neurocryptology has now become so important, I would like to say (although it is not entirely correct on my part) that the theoretical and methodological program for deciphering brain codes of psychic phenomena (primarily SR phenomena) was widely deployed from the standpoint of the information approach in my book that appeared more than 45 years ago. See: D. Dubrovsky. [4] This is mainly devoted to chapter 5. Pp. 241–358. The book is on the website: www.dubrovsky.dialog21.ru.

6 One of the ways of forming personal and interpersonal invariants of the visual image and the corresponding neurodynamic carriers (using the principle of system isomorphism) was elaborated in detail in paragraph 5 of Chapter 5 of the abovementioned book [4] (see Pp. 284–300). Here, the formation of the interpersonal invariant of any SR phenomenon in general was also considered.

7 The substantiation of this proposition and the detailed phenomenological analysis of the structure of SR are contained in: Dubrovsky D.I. The problem of the ideal. Subjective reality. 2nd ed. augmented, Moscow, Canon+, 2002 (first edition: Moscow, Mysl, 1983); present on the site: www.dubrovsky.dialog21.ru.

8 The principle of isofunctional systems means that the same function or complex of functions can be reproduced on substrates different in their physical (chemical) properties. A model example: a natural tooth is removed, an artificial tooth is inserted. The function is the same, the substrate is different. On the path of this type of substitution, it is difficult to establish any limit. Many internal and external organs of man are now successfully prothetized. This also applies to individual components of the brain (for example, implanting an electronic chip in the brain of a paralyzed person, allowing him to mentally manage a wheelchair, etc.).
